# Evaluating the perceived implementation and impact of the chronic dispensing unit in the Western Cape

**DOI:** 10.3389/frhs.2026.1776038

**Published:** 2026-03-09

**Authors:** Ilona Matthew, Michelle Viljoen, Jane McCartney, Angeni Bheekie

**Affiliations:** School of Pharmacy, Faculty of Natural Sciences, University of the Western Cape, Cape Town, South Africa

**Keywords:** CFIR, chronic disease, chronic dispensing unit, impact, implementation, NCD, primary healthcare, RE-AIM

## Abstract

**Introduction:**

The research explores the perceived implementation and impact of the Chronic Dispensing Unit (CDU) within a South African primary healthcare system, with a focus on chronic disease management, using the RE-AIM (Reach, Effectiveness, Adoption, Implementation, and Maintenance) and CFIR (Consolidated Framework for Implementation Research) frameworks. Equitable access to healthcare and medicine is still a challenge; it demands long-term care and ongoing medical interventions. Introduced in 2005, the CDU in the Western Cape was designed to overcome the challenges in access by centralising dispensing and distribution of chronic medicines. Two decades after its implementation, its contribution is underexplored. This research evaluated the long-term performance and sustainability of the CDU using implementation frameworks.

**Method:**

A qualitative design was used, using virtual semi-structured interviews with purposively selected participants (*n* = 8) involved in the implementation and maintenance of the CDU. Interviews were analysed thematically. A deductive-inductive strategy was applied, guided by the RE-AIM and CFIR frameworks.

**Results:**

The CDU demonstrated substantial Reach and Effectiveness. It has refined operational processes and reduced patient waiting times. Challenges with data integration and the non-collection of medicine limit the CDU’s ability to inform clinical outcomes and long-term sustainability.

**Conclusions:**

The CDU is still an effective, well-integrated system that supports chronic disease management but is constrained by disconnected data systems. This study evaluated a large-scale health intervention that facilitated data-driven decision-making to monitor, evaluate, and report on evidence-based programmes addressing barriers to sustainment. Integrating two frameworks provided an assessment of a patient-centred intervention, granting insights into equity in access to medicine, to strengthen primary healthcare systems.

## 1 Introduction

The First National Burden of Disease Study in South Africa was conducted in 2000 (1). It was the country's earliest attempt to derive estimates from the burden of disease and highlighted key mortality indicators in South Africa. The study identified a quadruple burden of disease, with AIDS, chronic diseases, poverty-related conditions and injuries all appearing among the top causes of death. At that stage, Bradshaw et al. (2003) identified that life expectancy at birth was approximately 55.2 years: 58.5 years for females and 52.4 years for males (1). Non-communicable diseases (NCDs) accounted for 37% of the deaths. Cardiovascular disease was responsible for a higher proportion of female deaths (approximately 19%) than males (14%), with Stroke being the highest NCD for females and Ischaemic Heart Disease the highest for males. Diabetes mellitus, respiratory conditions and hypertensive heart disease followed this. Furthermore, in the Western Cape (WC), NCDs accounted for a significantly higher proportion of mortality (approximately 58%) relative to the national average (38%) (2).

The Second National Burden of Disease Study indicated that the quadruple burden persisted and generated trends in causes of death from 1997 to 2009 (3). It provided evidence of the underlying causes of mortality. It showed a decline in mortality attributable to HIV/AIDS and Tuberculosis, compared to NCDs, which emerged as the predominant contributors to mortality in South Africa. The Cause of Death report by Pillay-van Wyk et al. (2016) indicated that HIV/AIDS and Tuberculosis accounted for 34.3% of all adult deaths, and NCDs accounted for 64.9% of deaths in persons older than 45 years of age (4). The Western Cape Government Health and Wellness (WCGHW) established a policy framework to further guide chronic diseases management in the province, prioritising interventions for cardiovascular disease, diabetes, hypertension, asthma and epilepsy (5).

The rise in chronic disease cases led to increased workloads and subsequent bottlenecks in the pharmacy workflow at hospitals and primary healthcare (PHC) facilities, resulting in longer waiting times for patients to receive their chronic medications (6). The complexity of tackling chronic diseases in the WC region highlighted the need for a targeted approach to pinpoint and address fundamental operational issues. Overcrowding and inefficiencies in hospitals and community health centres (CHCs) created an urgency. The need for improved pharmaceutical service delivery was recognised, and an intervention was necessary to relieve pressure on PHC facilities and to optimise the accuracy of medicine dispensing and medication adherence, a priority within the pharmacy industry. A medicine dispensing service was established.

The first Chronic Disease Management strategy drew heavily on systems thinking. It incorporated capacity-building, role definition, adaptation, and multi-level coordination. This provided a stronger rationale for the ongoing development of integrated management of other chronic diseases. The process aimed to develop services at all levels of care, improve health outcomes, and reduce attendances, waiting times, and hospital admissions (7).

The Chronic Dispensing Unit (CDU) was purpose-designed to meet the WCGHW-mandated standards for preparing and distributing monthly chronic medication parcels to facilities (8, 9). Stable chronic patients were clinically identified and enrolled into the CDU programme (10). Patients receive the first month's supply at the health facility; thereafter, they can collect medication refills either at CHCs or decentralised collection points, prepared by the CDU, for up to a maximum of five months. Afterwards, they are required to consult with the doctor or nurse again. The CDU started operations in the Cape Town metropolitan area in December 2005, with a phased implementation across the province's health centres (6).

**Figure 1:**
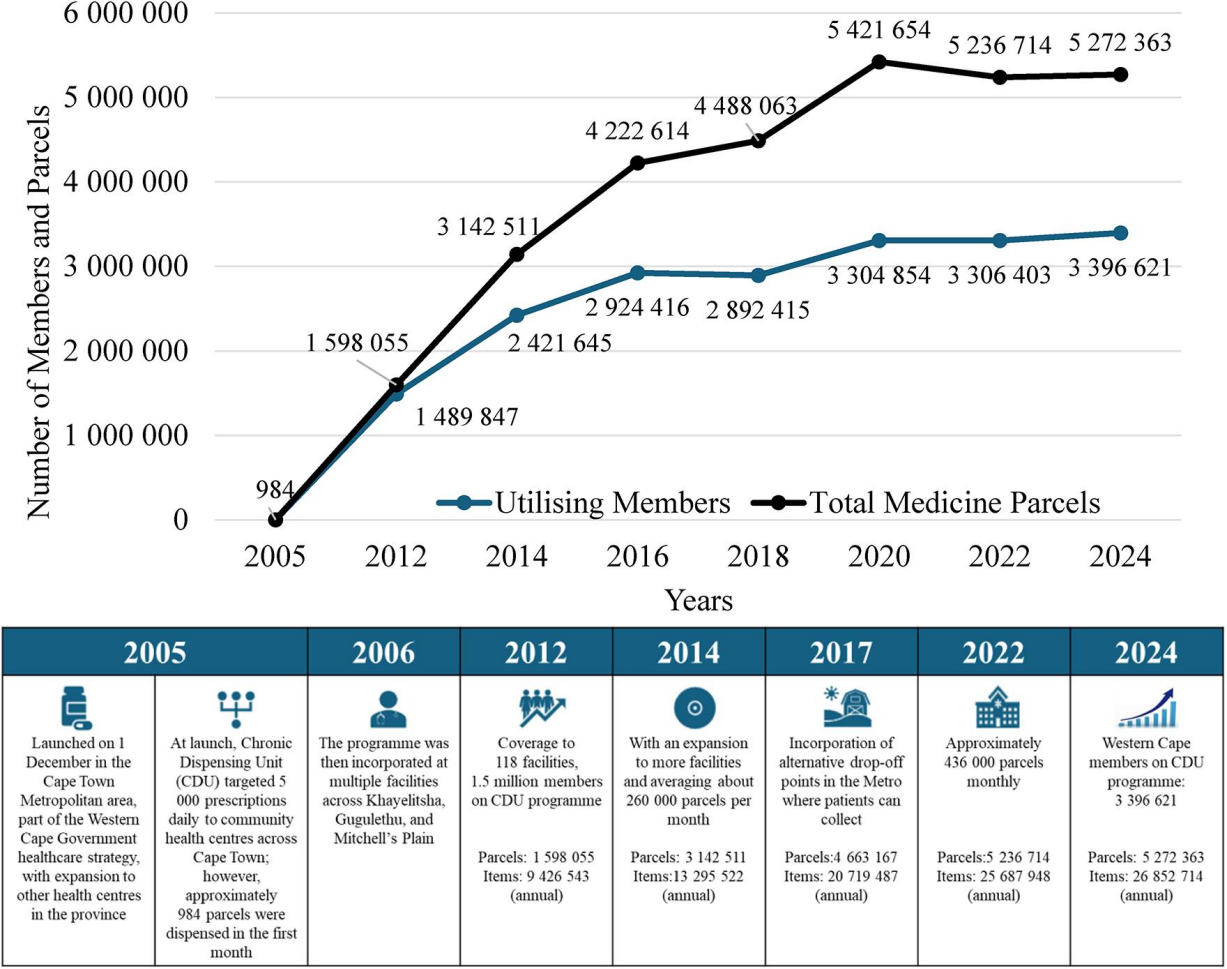
The CDU programme expansion, depicting the growth of members and medicine parcels over two decades, across the Western Cape province.

With an expansion over the years to include additional facilities on the programme across the province and rural communities, the programme has seen exponential growth in the number of registered programme members (patients), the number of medicine parcels packed (a member may receive more than one parcel), and the individual items dispensed per year for the entire WC province (Figure 1).

**Figure 2:**
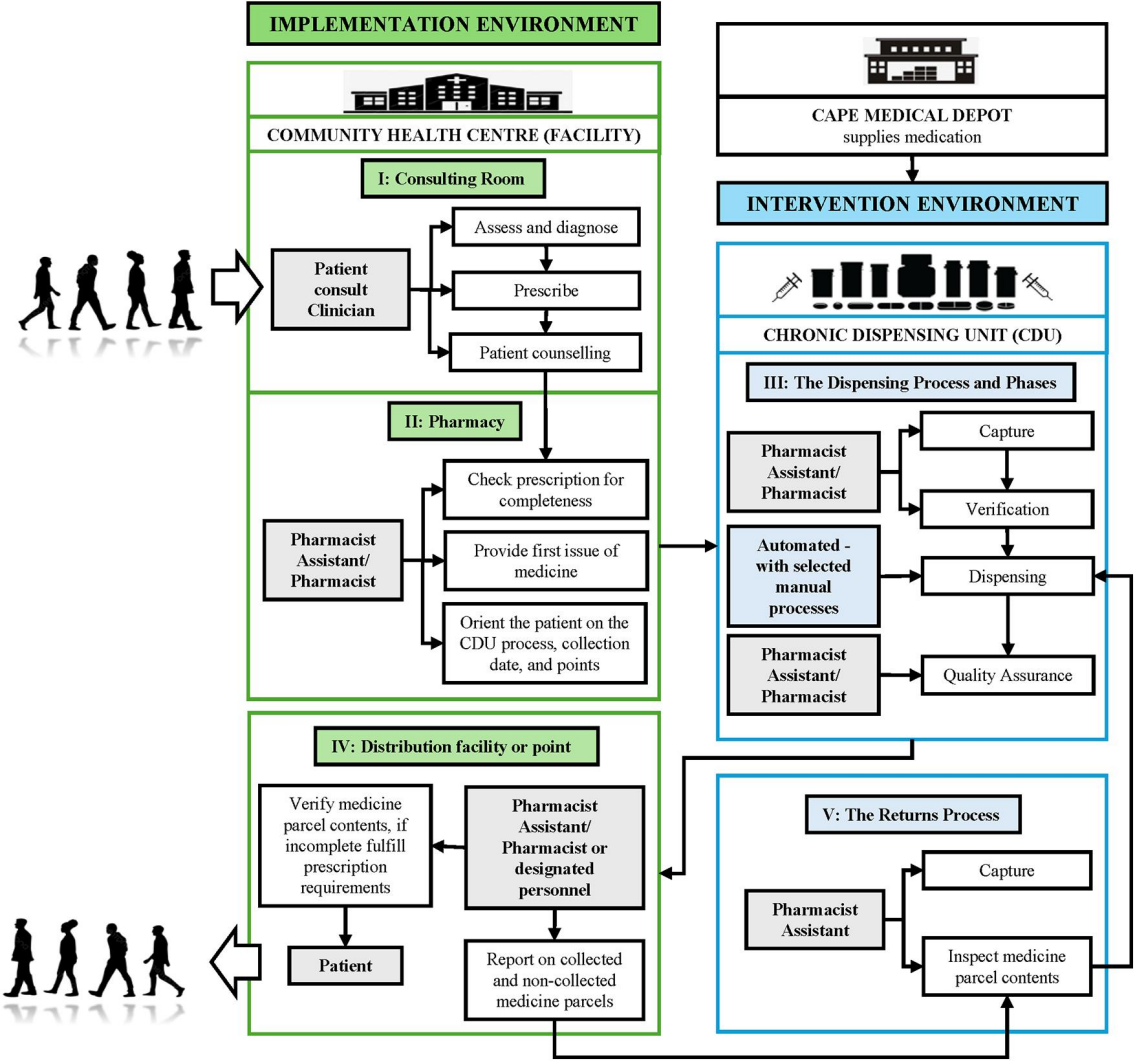
The CDU process indicates the flow of both prescription (in the intervention environment) and patient (in the implementation environment, where the impact can be seen), as adapted from Magadzire et al. (6).

Bulk medication is delivered from the WC Medical Depot to the externally contracted CDU service provider (10, 11). The service provider, which operates as an independent entity, receives, stores, and dispenses medicine at a separate centralised location in Cape Town (12). Paper prescriptions are physically collected from CHCs by the CDU for data capturing, and filled prescriptions are returned to decentralised distribution sites, either CHCs (hospitals and clinics) or designated points (clubs, churches, or community centres), where patients collect their medicine parcels (Figure 2).

**Figure 3:**
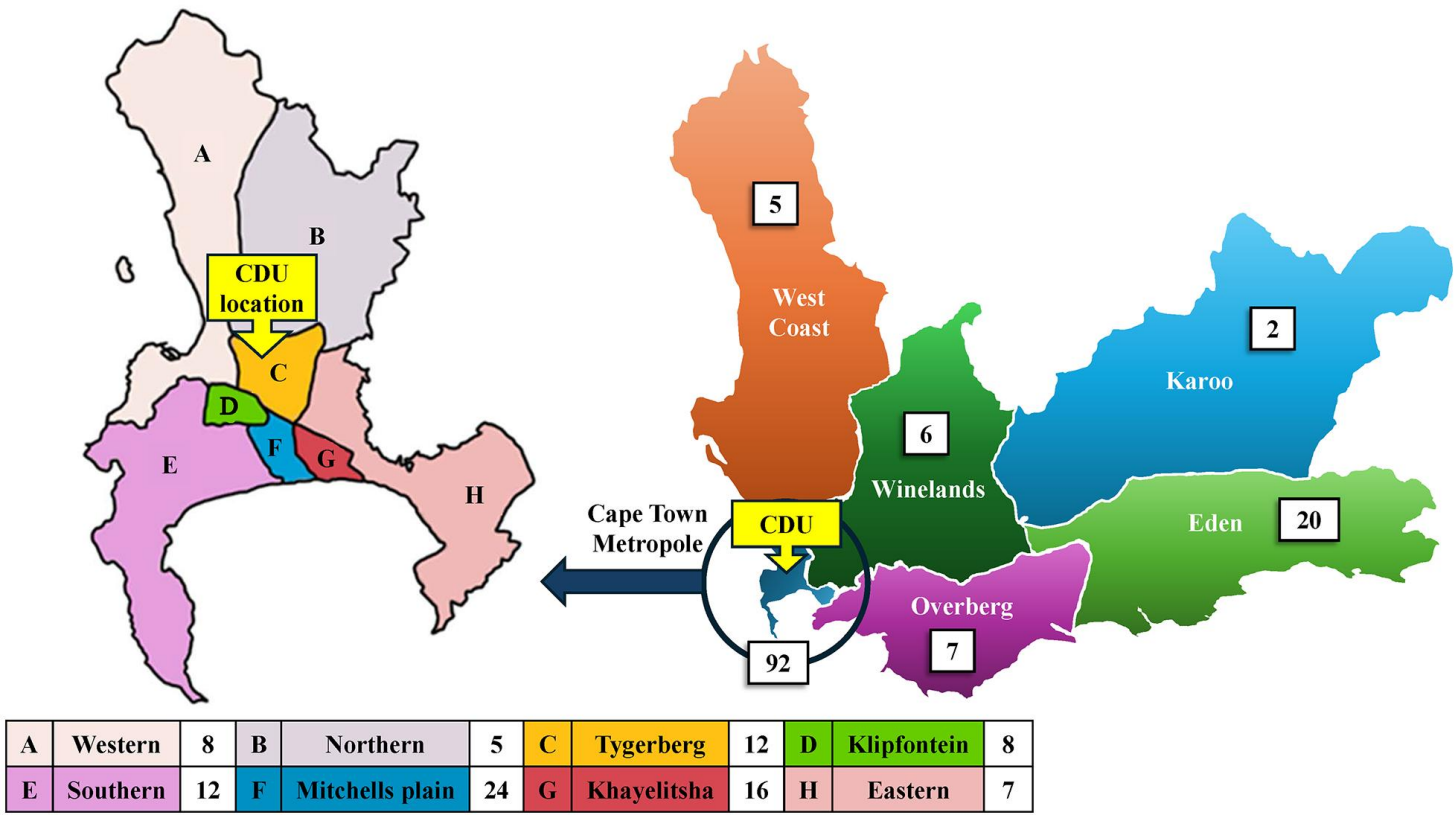
A map of Western Cape Province on the right, indicating the number of CHC delivery and collection points (excluding alternate points) that the CDU provides a service to, and an enlarged map of the Cape Town Metropole on the left, indicating its eight subdistricts.

Initially introduced to alleviate long waiting times and reduce congestion at public health facilities (6), the CDU programme has responded to exponential growth in patient numbers (Figure 1*)*, with scalable, semi-automated systems (10, 11). The number of drop-off points grew from the Cape Town Metro's eight subdistricts to the rest of the province's rural subdistricts, where patients can collect their medication from 134 hospitals, clinics, chronic clubs or alternate collection points (9) (Figure 3). The CDU has now been in operation for two decades (Figure 3).

### 1.1 Problem Statement

The disease profile for South Africa is complicated by inequities across its nine provinces, where variations in mortality reflect underlying socio-economic disparities (13). Research shows that poorer communities suffer more from illness and disability than wealthier groups. Even diseases that were previously linked to wealth, like NCDs, are now increasingly affecting poorer communities (14, 15). Recently, Statistics South Africa reported that deaths due to major diseases such as cardiovascular disease, diabetes, cancer and chronic lower respiratory disorders increased by 58.7% over 20 years (16). With a steady increase in NCDs, Mayosi et al. (2009) indicated there is constant competition for equitable access to health and medicine, with the remainder of the quadruple burden of disease (17). Access remains a challenge, even more so when trying to manage the increasing change in disease profile, and associated long-term chronic care and continuous medical interventions, especially in disadvantaged communities. Interventions and action plans to minimise the rising mortality rates became necessary to mitigate population-level risks associated with NCDs (13, 18) The CDU was one of the pharmaceutical interventions implemented to overcome the challenge of access to medication. However, the implementation of health policies, from design to routine practices, can be a challenge if they fail to achieve the intended outcome.

### 1.2 Research question

How has the implementation of the CDU influenced equity of access, healthcare delivery, and sustainability of chronic medication services across the WC?

Secondary questions:

To what extent has the CDU achieved equitable reach to patients across the districts within the WC?
What are the key facilitators and challenges that influenced the CDU’s adoption, implementation and its sustainability of the CDU?
How has the CDU influenced the continuity of medicine supply among patients?
What systemic gaps have limited the CDU’s ability to inform public health planning?

### 1.3 Aim

The research aimed to evaluate the perceived implementation and impact of the CDU as a PHC system intervention, with a focus on access to chronic medication and service delivery outcomes over two decades in the WC.

## 2 Method

### 2.1 Study design and framework

**Figure 4:**
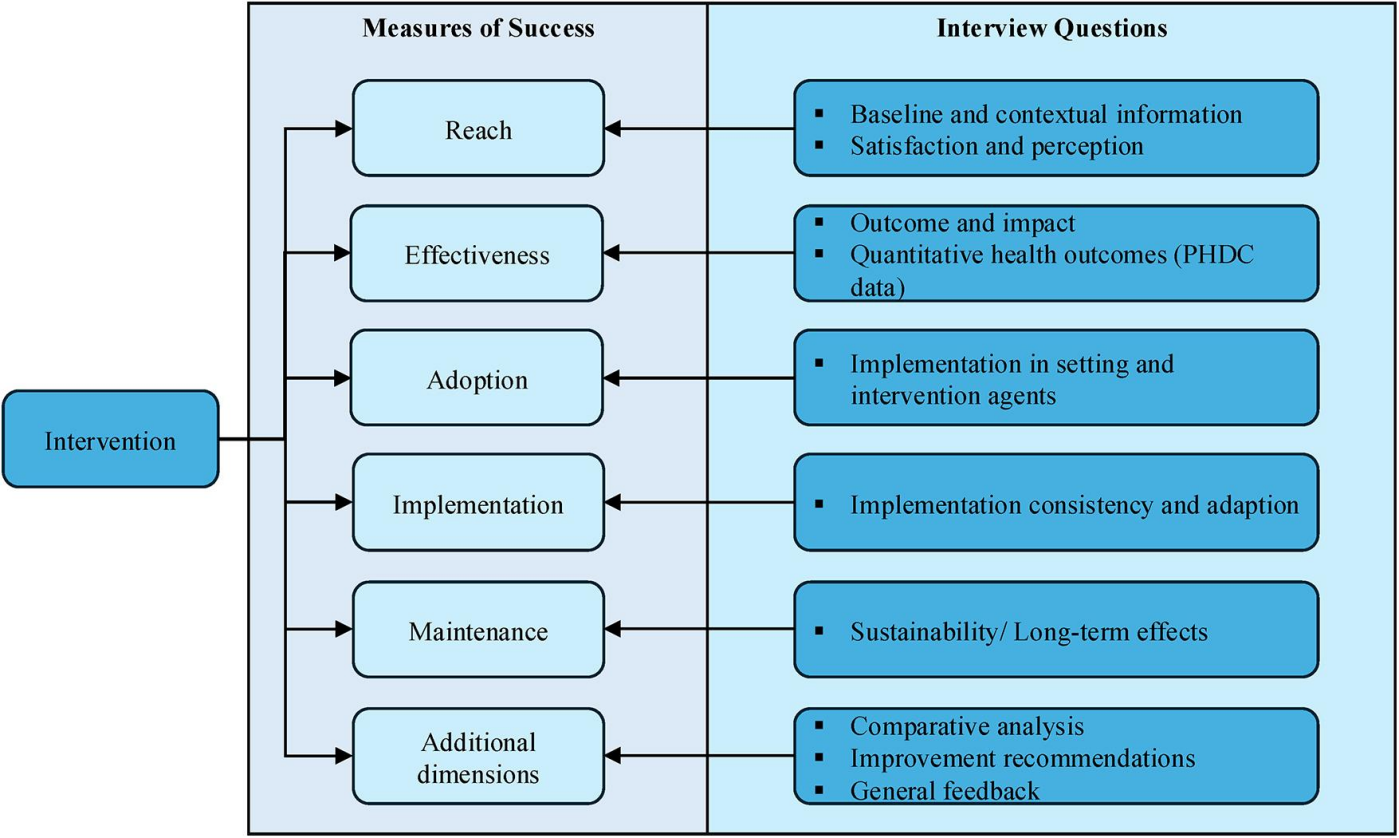
Illustration of the RE-AIM framework used to guide the interview questions.

A retrospective qualitative design was employed to evaluate the performance of the CDU intervention, using the combination of RE-AIM (Reach, Effectiveness, Adoption, Implementation, and Maintenance) (19, 20) and CFIR (Consolidated Framework for Implementation Research) (21) frameworks. Semi-structured, virtual interviews were used to explore stakeholder perceptions, experiences and insights. This method provided insight into the intervention's facilitators and the challenges associated with addressing access to chronic medication. The dual framework informed the design of interview questions and guided thematic analysis. To measure the perceived implementation and impact of the CDU, the RE-AIM (focused on what happened) and CFIR (explained why it happened) constructs were used, both of which are employed as effective models for measuring evidence-based programmes (22). For a programme like the CDU, where policy translates into practice, the dual frameworks ensured a comprehensive evaluation of the unit's performance. It included the intervention's implementation outcomes and the contextual factors, including its long-term impact, sustainability, and implementation effectiveness (23, 24). The RE-AIM framework guided the selection of interview questions with integration of the CFIR domains (Figure 4).

By combining the RE-AIM and CFIR frameworks, the study generated an understanding of both the outcomes of the intervention and its implementation (22–24). RE-AIM questions focused on the extent to which the CDU has served the target population, the adoption of the CDU model, the evaluation of the quality and consistency of service delivery, and whether the benefits of the CDU have been sustained over time. Additionally, the CFIR framework was employed to assess the inner and outer settings, the process, and both intervention characteristics and individual characteristics, informing data collection and analysis, and evaluating how the CDU has evolved. The participant responses from the semi-structured interviews were analysed and documented using thematic analysis and quotations.

### 2.2 Setting, sampling, size and participant inclusion

Setting: The WC health system, with a mix of urban and rural service delivery by WCG officials and outsourced service providers, offered a rich context to the CDU programme's implementation.

Sampling: This research drew on multiple perspectives from these information-rich individuals to gather in-depth data (25). A purposive sampling method was used to recruit all interview participants, with both direct and long-standing involvement in the health programme across the WC, combining institutional memory and operational experience.

Inclusion criteria: Participants were selected to ensure alignment with the objectives of the study, with direct impact on the programme's policy and implementation. All WCGHW participants have held multiple positions across PHC facilities, operational district management, pharmaceutical services, and provincial-level administration and management. Participants' initial roles as frontline workers at the health facility level during programme implementation have morphed into managerial and strategic roles over the years. Their experience across facility, district and provincial levels provided a holistic view of operational and strategic processes, reflecting both policy-level decisions and frontline realities. This ensured representation from multiple health system levels. Participants were selected from both metropolitan and rural districts, and included both WCGHW and outsourced service providers. Participant interviews were requested and approved through the WCGHW research unit. Recruitment was conducted via departmental referrals and direct invitations to eligible personnel. Participants consented voluntarily.

Size: A sample size of approximately 10 participants was anticipated. Given the specialised nature of the participants, only a small number were expected to meet all the criteria, given their expertise, multiple positions, experience levels, and years of service. Eight participants were interviewed, collectively providing a comprehensive range of insights into the implementation and the operational dynamics, allowing for information-rich narratives to explore the perceived implementation, integration, and impact of the CDU programme across different levels. The sample size was deliberately structured to include diverse perspectives from both metropolitan and rural contexts, WCGHW and outsourced service providers. Participants filled roles across the pharmaceutical, clinical, and administrative domains. Eight participants were deemed sufficient, as interviews reached thematic saturation and yielded recurring insights across different health system levels. Participant data to contextualise the study findings are presented in Table 1.

**Table 1:** Demographics indicating the profile and experience of the study participants.

Participant number	Gender	Age range (in years)	Rural/Urban	Participant affiliations (and level)	Years of experience across departments
1	Female	50–65	Urban	WCGHW (operational management)	30+
2	Female	50–65	Urban	WCGHW (senior management)	25+
3	Male	40–50	Urban and Rural	WCGHW (senior management and policy)	25+
4	Male	50–65	Urban	CDU Service Provider (senior management)	20+
5	Female	50–65	Urban	WCGHW (senior management and policy)	40+
6	Female	50–65	Urban and Rural	WCGHW (policy)	40+
7	Female	50–65	Urban	WCGH, CDU Service Provider and Private industry (operational management)	30+
8	Male	30–40	Urban and Rural	WCGHW (senior management)	15+

WCGHW, Western Cape Government Health and Wellness; CDU, Chronic dispensing unit, an independent entity that dispenses medicine at a separate centralised location.

### 2.3 Exclusion criteria

All PHC facilities outside the WC province were excluded from the research. All persons with no direct involvement in the CDU programme or its implementation and maintenance were excluded from the interviews.

### 2.4 Data sources and collection

Data were gathered through semi-structured interviews, as guided by the RE-AIM and CFIR frameworks. A pilot interview participant, who met all the established inclusion criteria, was interviewed under the project supervisor's supervision. The pilot interview followed a semi-structured guide and was used to improve three questions and improve the interview flow; however, the results were not included in the final analysis. To further minimise bias, all interviews were virtually audio-recorded and transcribed. Each interview followed the semi-structured guide. Participants were invited to partake, and written consent was obtained prior to the interviews. The primary researcher conducted the interviews, with no prior personal relationship with any of the participants. This ensured neutrality, and minimised potential bias during data collection. The researcher remained cognisant of the dynamics during interviews, taking a non-leading, impartial position, to promote open and authentic responses. This approach enhanced the credibility and trustworthiness of the findings. To validate the primary data, each recording was independently reviewed by an external reviewer who had no direct influence on the study design or data collection. The transcripts were cross-checked and verified for accuracy by the same reviewer. The last round of analysis was then discussed and further iteratively refined by the entire research team, consistent with recommended qualitative methods for applying the frameworks (26).

### 2.5 Data analysis

#### 2.5.1 Framework

The RE-AIM and CFIR frameworks guided the thematic analysis approach and structured the interpretation of the data. A thematic analysis was conducted by becoming familiar with the data, using codes guided by the two frameworks, and following the phases: familiarisation, coding, identifying themes, by reviewing potential themes, defining and naming the themes, and producing the report (27). Transcripts of audio recordings were independently verified and analysed. Together, these frameworks enabled the dual-level analysis.

#### 2.5.2 Framework integration

The hybrid technique provided in-depth insight into implementation outcomes (RE-AIM) and contextual factors (CFIR) that influenced the CDU programme. In this manner, RE-AIM was compared with CFIR to identify how context shaped the outcome.

#### 2.5.3 Coding type and process

A deductive-inductive coding approach was used. Initial codes were predefined using RE-AIM and CFIR concepts and served as the foundation for the structured codebook. Inductive coding identified emergent subthemes within the CDU context. The operational definitions describe how each construct was expressed within the context of the CDU (Table 2).

**Table 2:** The codebook for thematic analysis, with a joint display matrix of integration of RE-AIM and CFIR frameworks domains.

RE-AIM Domain (Deductive)	Inductive Theme (from interviews)	CDU operational context (Definitions of the inductive theme)	CFIR Domain (Deductive)	CFIR construct (Deductive)
Reach	Expanded geographic access	The extent to which CDU improved accessibility for patients with chronic conditions	Outer Setting	Patient Needs and Resources
	Patient-centred care	The quality of the intervention design, which prioritises the patient	Intervention Characteristics	Design Quality
	Equity in access	Capacity and layout at the facility level	Inner Setting	Structural Characteristics
Effectiveness	Improved service delivery	Perceived reliability of the CDU model in improving care	Intervention Characteristics	Evidence Strength and Quality
	Clinical governance	Systems for monitoring and feedback loops	Process	Feedback and Evaluation
	Data-driven decision-making	Data systems supporting evidence-based management	Intervention Characteristics	Complexity and Data Systems
	Perceived patient satisfaction	Improved patient experience when collecting chronic medication	Individual Characteristics	Beliefs and Self-Efficacy
Adoption	Gradual buy-in	Organisation receptivity and staff commitment	Inner Setting	Culture and Implementation Climate
	Training and support	The quality of staff training and ongoing support structures	Process	Engaging and Executing
	Policy alignment	Alignment with provincial and national policy directives	Outer Setting	External Policies and Incentives
Implementation	Operational challenges	Infrastructure or resource constraints during implementation	Inner Setting	Structural Characteristics
	Innovative workflows	The integration of new workflows into existing systems	Process	Executing and Reflecting
	Service provider dynamics	Coordination among WCGH staff and service providers	Process	Planning and Contract Management
	Contractual evolution	Adaptations made over time to improve service level delivery	Process	Continuous Improvement
Maintenance	Sustainability and integration	Long-term feasibility and the cost-efficiency of CDU	Intervention Characteristics	Cost and System Maturity
	Financial viability	External budgetary limitations	Outer Setting	Funding Constraints
	Recommendations	Forward-looking planning to institutionalise CDU	Process	Strategic Planning

#### 2.5.4 Codebook approach and development

The codebook was developed prior to any formal coding to guarantee consistency and reproducibility. It contained the domain for the framework, the concept, the CDU context, and the codes. The codebook was iteratively refined as inductive insights emerged during analysis. Complete transcripts were uploaded to ATLAS.ti platform and coding information were captured using the ATLAS.ti platform. The primary researcher independently coded the transcripts using the agreed codebook and incorporated inductive sub-themes that emerged from the interviews onto the ATLAS platform. Credibility was enhanced by the involvement of an independent reviewer, who assessed the verbatim transcripts and the initial coding outputs. The reviewer then evaluated the assigned codes, emerging themes and subthemes, confirming the accuracy of the research participants' accounts. Differences were identified and resolved using structured, iterative discussions. To improve dependability, the independent reviewer examined the consistency of the codes across the transcripts, ensuring uniform coding with the evolving codebook, code refinement, and theme development. Reliability was strengthened through independent verification of codes across transcripts, alignment of the RE-AIM and CFIR frameworks, and adherence to the predefined constructs. Together, the measures demonstrated a rigorous and transparent analytical process. Furthermore, agreement was then reached through structured discussion with the research team until consensus was reached.

### 2.6 Interpretation of themes

#### 2.6.1 Deductive themes

All RE-AIM domains (*Reach, Effectiveness*), CFIR domains (*Intervention characteristics, Inner settings*), and CFIR constructs (*Patient needs and Resources*) were pre-defined from the framework. This provided a top-down structure.

#### 2.6.2 Inductive themes

The context-specific themes listed (for example, *Expanded access, Gradual buy-in, Operational Challenges*) were inductively derived from interviews. They represent contextual expressions of the deductive constructs within the CDU intervention.

#### 2.6.3 Joint display and integration

To integrate the findings, the results mapped the RE-AIM deductive theme to the inductive code, which contained themes, inductive codes, and quotes. This allowed convergence between qualitative and quantitative data, showing how and why the CDU achieved its outcomes across the dual frameworks, and therefore reinforcing the validity of the analysis.

### 2.7 Ethical considerations

Ethics approval, from the University of the Western Cape's Biomedical Science Research Ethics Committee (BMREC), was granted in November 2024. The BMREC ethical clearance number is BM24/10/18. WCGHW clearance was received on 30 January 2025 to conduct participant interviews, under number WC-202501_002. Participant participation was voluntary; they could withdraw at any time. Written consent to participate was requested and obtained before the interviews, which were audio-recorded for transcription and analysis. Participants' identities were protected. To maintain participants' confidentiality, numbers were used in Table 3*.*

**Table 3:** Integrated implementation interview outcomes based on the RE-AIM framework to show convergence with CFIR.

Re-AIM domain	Inductive code RE-AIM with CFIR	Qualitative insights: deductive themes based on the collective perspectives of the interview participants	Inductive Quotes [participant number]
REACH Theme: expanded access	Expanded geographic access and Patient Needs & Resources	CDU enabled expanded access to medicine and alternative collection points closer to patients’ homes, in rural areas, and for vulnerable groups (e.g., the elderly, seasonal workers). Overcrowding and inefficiencies created urgency. CDU addressed long-standing issues such as travel burdens, long queues, and monthly work absenteeism.	Decentralisation ‘We deliver directly to old age homes…seasonal farm workers…mobile clinics.’ [4] ‘It's about providing patient care, closer to home’ [6] ‘You don't want patients to drive to X Hospital, for example, for their repeat medication. They can get it much closer to their homes now.’ [1]
	Patient-centred care and Design Quality	Participants highlighted reduced travel burden and improved convenience, particularly for patients with chronic stable conditions. CDU’s design evolved but requires improvements in data systems and medicine parcel management.	Innovation ‘There's a CDU window dedicated to that (collections), and that made a lot of sense because you could turn over quite a large volume of clients rapidly at the facilities.’ [8] ‘Some facilities have clubs, and patients come to the club for advice, dietary education, and so forth when they collect their medication.’ [5] ‘There is a separate queue for CDU patients.’ [2] ‘At some facilities, they may have … a CDU window, and all patients collecting their parcels know that window.’ [5]
	Equity in access and Structural Characteristics	CDU supported convenient collection points, mobile clinics, and innovative staff solutions at facilities, including separate CDU windows, lockers, and club models.	Equity and innovation ‘We also have collection boxes, it's pick-up points, and people get a code, and they can go and collect their medicine.’ [1] ‘During the COVID pandemic, we were trying to aim for minimal patient contact; we combined CDU with home deliveries.’ [8] ‘We wanted patients to receive their medicines within a shorter waiting time.’ [7]
EFFECTIVENESS Theme: Improved service delivery and perceived patient outcomes	Improved service delivery and Evidence Strength and Quality	Automation and decentralised dispensing increased throughput, facilities reported shorter queues and faster medicine collection for CDU patients. Participants consistently affirmed CDU’s effectiveness in improving access, reducing waiting times, and enhancing rational use of medicines.	Efficiency gains ‘No human can dispense at that rate, like a machine does. And with the parcel already being made up, when the patient arrives at the facility, it's there, and it's ready.’ [2] ‘We can concentrate on better patient care’ [7] ‘Medicine collection is merely a logistical event…and pre-packed medicines opened the possibility to facilitate access.’ [8] ‘They would not have coped in terms of the size of their pharmacies in service if not using automated systems.’ [1]’
	Clinical governance and Feedback and Evaluation	CDU enforced formulary compliance and adherence to rational prescribing practice, flagged irrational prescribing, and enhanced patient safety by vetting and rejecting problematic prescriptions. Contracted service providers played a central role in operational success	Governance and safety ‘They’ve really improved prescribing practices… highlighting problems like adverse drug interactions.’, ‘We’ve also picked up irregular prescribing practices and compiled a list of medicines which should not be prescribed together.’ [1] ‘We can monitor our standard of care; we can stick to the formulary.’ [2] ‘Clinical rejections are processed on scripts that are queried, such as overdosage, inappropriate (medicine) combinations, formulary diagnosis not allowed for chronic use.’ [4]
	Data-driven decision-making and Complexity & Data Systems	Valuable data are generated, and enhanced data availability enables monitoring of prescribing patterns, medication use, and adherence. There are concerns that data integration with broader health systems within the WCGH remains limited. A high level of complexity is noted in automation, data systems, and prescription vetting, and the service provider highlighted the operational intricacies.	Data utility ‘We can look at prescriber behaviour, even at a facility level, for irrational medicine prescribing or adherence to standard treatment guidelines.’ [5] ‘We now have proper data… to support referral patterns, disease profiles, and patient demographics.’ [5] ‘We can track things like…the number of prescriptions, deliveries, upliftment, non-collections and the financial impact of uncollected medicine.’ [2] ‘System integration will work…clinics and hospitals may have the data, but the systems do not talk to each other.’ [7]
	Patient satisfaction and Beliefs & Self-Efficacy	Removing chronic patients from acute queues improved service delivery; most participants rated CDU highly (scores between 6 and 9, out of 10), citing a (perceived) improved patient experience and reduced facility congestion. Leadership and commitment from key individuals were crucial. Most participants expressed a strong belief in CDU’s value	Improved patient experience ‘Previously, patients had to take a day off from work, sometimes even unpaid, and stand in long queues. Sometimes they even had to return the next day.’ [7] ‘There is no longer that patient waiting time for people that are working, you don’t have to take a day’s leave.’ [5] ‘We started off with facility-based collections; they still came into the facility, but they did not have to go via reception. They came in with their card, with your folder number on it. And then we would issue a medicine parcel, like a quick pick up.’ [8] ‘It's much easier for the patients now, but it also declutters the facility, and these patients are not part of the normal queue within the pharmacy. So, it helps the other patients who are more acutely ill to shorten the queues.’ [1]
ADOPTION Theme: Gradual buy-in and integration	Gradual buy-in and Culture and Implementation Climate	Initial resistance from staff and management indicated the presence of cultural barriers, with low enrolment in the early phases due to unfamiliarity and perceived complexity. Initial resistance gave way to acceptance and grew with demonstrated benefits. Over time, CDU became an integral part of routine operations	Initial resistance ‘When we implemented, things didn’t go as smoothly. We expected thousands of prescriptions in the first month… got under 1000. It is something that has never been done, so we knew that there had to be a change in behaviour.’ [2] ‘It was resistance to change, and the change was not just at the local level; it was all the way up.’ [5] ‘At facilities, staff felt like we were taking work away from them.’ [7] ‘There was uneasiness from clinicians, because you basically sent your patient away for months. But the platform understands the value, and there is a real buy-in.’ [8]
	Training and support and Engaging and Executing	Structured support implementation teams, brochures, and liaison officers helped educate staff and patients. Implementation teams, liaison officers, and oversight facilitated communication and problem-solving.	Training impact ‘We had unqualified support from the HOD at the time.’ [5] ‘We had to provide training because patients were unfamiliar with the concept of a chronic dispensing unit. We conducted training sessions for the staff and gathered their suggestions on how to implement.’ [2] ‘Ongoing training is provided by the service provider. Client Liaison Officers (CLOs) are allocated certain clinics …they offer individualised training specific to the needs or challenges of the facility.’ [7] ‘CLOs continually train prescribers, train the personnel, and provide constant feedback.’ [4]
	Policy alignment and External Policies and Incentives	Over time, CDU became an integral part of routine operations and was widely accepted as standard practice throughout the province. National initiatives, such as the Central Chronic Medicines Dispensing and Distribution (CCMDD) programme and the National Health Insurance (NHI), may influence CDU’s trajectory.	Policy integration ‘Yes, the programme is without a doubt now part of routine operations.’ [4] ‘There was buy-in. It took time. It took several years for all this to happen.’ [5] ‘It is an inseparable part of what we do. I do not think we will survive if we pull it (CDU project) out.’ [8]
IMPLEMENTATION Theme: Operational challenges and innovations	Operational challenges and Structural Characteristics	The rollout faced several challenges, including space constraints, prescription chart formats, prescription errors, issues with referral pathways, and uncollected parcels. A change in service providers experienced challenges due to a lack of foresight in data handover.	Rollout issues “We also found that space was an issue… boxes full of prescriptions coming to the facilities, and facilities did not have sufficient space.’ [2] ‘Initially, we saw quite serious dispensing errors… We've got massive amounts of uncollected parcels. It's also more work to disassemble a patient's medicine parcel than it is to dispense it.’ [1] ‘At facilities, the issue was ‘change management’ and how patient flow was being managed.’ [6] ‘There were challenges from both sides, both service provider and at the facilities.’ [7]
	Innovative workflows and Executing and Reflecting	Facilities varied in their readiness, with some lacking space, staff, or necessary systems. CDU integration required infrastructure changes (e.g., shelving, separate dispensing areas or windows). Staff adapted workflows and innovated locally, showing confidence in managing CDU-related tasks. Dedicated teams supported facilities during rollout. Facilities adapted with CDU collection windows, separate queues	Workflow redesign ‘There have been quite a lot of innovative solutions at different facilities.’ [5] ‘We had to redesign patient flow… to accommodate CDU.’ [6] ‘We had to change our prescription charts to indicate where the person must go and whether it's delivered at another site for collection. That had to go on the prescription.’ [2] ‘There are HIV adherence clubs… their requirements, compared to a local facility, are different.’ [7]
	WCGH staff and Service provider dynamics and Planning and Contract Management	Transitions between earlier service providers revealed issues with software compatibility; phased handovers and continuous improvement were key strategies. Feedback loops, data analysis, and contract reviews informed ongoing adjustments but created unrealistic performance expectations.	Contractual Management ‘There needs to be a good partnership. They (service provider) cannot dispense in isolation. They’ve got to have that good partnership with us. That is important.’ [1] ‘The service design has to fit into a very narrow space according to the rules, and everything built into the management of the service provider prohibits us from really moving forward with the CDU.’ [3] ‘There must be penalty clauses. You know the system itself is not without its inherent risks.’ [5]
	Contractual evolution and Continuous Improvement	Penalty clauses and phased handovers were introduced to enhance accountability and continuity.	Contract learnings ‘It has translated into contract management … specifically, improving contract management, penalties, and related aspects that weren't initially so robust.’ [6] ‘Through the years, it has smoothed out by extending the length of the contract of the service provider. It creates much more continuity in service. The service is fantastic, but we still have a ‘paid for service’ which is budgeted for.’ [8]
MAINTENANCE Theme: Sustainability and system integration	Sustainability and integration and Cost and System Maturity	CDU is now considered essential, with no feasible alternative due to its volume and automation. Contractual penalties and performance matrices shape maintenance.	System maturity ‘It is sustainable, because it has improved year on year.’ [2] ‘The process has matured over the last 20 years, and there have been a lot of learnings along the way.’ [6] ‘It’s embedded in the way the department functions… impossible to re-source it. One of the success factors is the dedicated teams, from both the department and the service provider side.’ [5] ‘We do not have the capacity within the Western Cape Government pharmaceutical sector to dispense the volume of prescriptions without the support of the CDU now. It is not a nice-to-have. It's absolutely essential.’ [1] ‘The penalty matrix can hinder the sustainability (of the project). At the end of the day, the service providers are privately owned, and it's a business.’ [7]
	Financial viability and Funding constraints	Participants emphasised the need for cost-efficiency. The collection of medicines and future service fees was a concern.	Financial pressure ‘The biggest risk is if it becomes unaffordable.’ [1] ‘It all comes down to finances, and with better budget control, there will be a balance in how we approach the complete system.’ [3] “We are spending money to dispense medicine which is not collected.” [2] ‘We have a finite budget, and we have to manage that budget appropriately. Funding is always a constraint.’ [5] ‘Facilities do not always return medicine parcels, or do not return them on time, so that has a direct impact on the budget.’ [7]
	Recommendations and Strategic planning	(i) Integration of CDU data with e-prescribing systems, (ii) Better patient selection criteria to reduce non-collected parcels, (iii) Enhance contract management and improve collaboration with service providers, (iv) Decentralising financial and operational control to the districts, and (v) Establishing stronger public-private partnerships. (vi) Multi-month dispensing to reduce costs (vii) Facilitate access (in future) through micro-providers	‘The department needs a bona fide E-prescribing system integrated with the pharmacy management system.’ [1] ‘I would put management into the hands of the integrated service managers.’ [3] ‘Improve the relationship with the service provider.’ [4] ‘Better health record management. System integration will work wonders, integrating the service provider, the hospital, and the clinic.’ [7] ‘Risk-benefit is a huge consideration for the number of patients we serve; multi-month dispensing up to six months shows immense value.’ [8]
SUMMARY of perspectives		Strengths: Patient-centred design, data-driven governance, and a predominantly collaborative rollout. Challenges: Financial sustainability, data integration, and managing expectations. Overall: Highlighted operational complexity, with the need for mutual understanding and collaboration. Emphasised access, governance, patient safety, data utility, and systemic integration.	

## 3 Results

Table 3 represents the key integrated qualitative findings of the research, whereby the RE-AIM domains are mapped against the CFIR domains, as outlined in Table 2*.* Each cell represents a theme that links to deductive constructs, along with inductively generated themes that emerged from the interviews. Illustrative quotes demonstrate participants' experience and perceptions. Collectively, Tables 2 and 3 provide a comprehensive depiction of how the contextual and organisational determinants shaped the reach, adoption and sustainability of the CDU intervention. Key findings from interviews provide an overview of the strategies applied, based on the challenges experienced and how they were addressed.

### 3.1 Key findings from the RE-AIM framework

Reach. The CDU expanded access and decentralised collection points. It also reduced travel time and congestion at hospitals and clinics.

Effectiveness. Service delivery improved, with enhanced clinical governance. Perceived patient satisfaction increased due to reduced waiting times.

Adoption. Initial resistance was reduced, with gradual acceptance, and training was provided.

Implementation. The rollout faced logistical, infrastructural, and contractual challenges, with adaptive workflows. Service provider coordination and governance have evolved over time.

Maintenance. It is now integral to operations. However, non-collection of medicine parcels remains a challenge. Rigid contracts threaten long-term viability.

### 3.2 Key findings stemming from the CFIR framework

Inputs. Policy, human resources, and infrastructure were adequate to establish the CDU.

Activities. Centralised chronic dispensing and courier distribution reduced facility congestion.

Outputs. Increased medication distribution and enrolment were achieved.

Short-term outcomes. Reduced waiting times and perceived patient satisfaction were realised.

Long-term outcomes. Improved chronic disease control and health equity remain underachieved due to limited integration of dispensing and clinical data systems.

## 4 Discussion

Subsequent to the First Burden of Disease Study, the early 2000s decline in the population growth rate was due to the HIV epidemic (28), with a prolonged recovery supported by strong public health interventions (29). Statistics South Africa recently indicated that the South African life expectancy has demonstrated an upward trajectory over the last two decades. There was a temporary setback during the COVID-19 pandemic, but the country has shown a steady recovery phase (30). In the WC, PHC is essential to managing the burden of chronic diseases. The CDU programme promotes the co-management of HIV/AIDS and NCDs to reduce the strain on tertiary health services. The intervention environment, as depicted in Figure 2, makes a significant contribution to the implementation environment by improving the quality of service to stable chronic patients by introducing collection points closer to homes, through E-lockers, chronic clubs, and in some areas, wellness hubs (9, 31). According to the WCGHW, these additional innovative measures improve working conditions for personnel across all healthcare centres by reducing patient congestion. The decentralisation is aligned with the World Health Organization's plan on strengthening PHC in low- and middle-income countries, being more people-centred and through medication delivery of integrated health service models (32).

### 4.1 Interpretation of key findings

The programme was implemented to reduce long waiting times at hospitals and PHC clinics, alleviate overcrowding, and improve inefficient workflows. The dual framework analysis reveals consensus of all participants on the CDU's potential to provide equitable access to medicine for patients with chronic conditions in the WC (Table 3). To note, Western Cape CDU research is rare and largely limited to Magadzire et al. (2015) (6). However, several studies on the national Centralised Chronic Medicine Dispensing and Distribution (CCMDD) programme have been conducted, in which the CDU served as a reference model for the national initiative. CCMDD research largely focuses on process, access, patient experience and operational feasibility.

Improved Access and Reach: CDU member data show a steady rise in enrolment across the Western Cape Province (Figure 1). Participants indicated that the programme improved equitable access to chronic medication, and they noted that patients valued the mobile clinics, CDU windows, and lockers, particularly for rural and vulnerable communities. Alternative collection points within the community meant patients no longer had to wait at CHCs, thereby improving the patient experience. Magadzire et al. (2015) corroborate the interview participants' feedback regarding access and efficiency, and note process outcomes, but not long-term clinical outcomes (6). Collecting medication at a more convenient location improves patient adherence and ultimately reduces the burden on the health system (9). These findings align with evidence from other countries that private partnerships with decentralised medicine collection points, and patient-centred models personalised to the needs of the person and community, improve medicine compliance (33, 34). Similarly, in sub-Saharan Africa, Tanzania and Uganda are exploring the decentralisation of chronic disease management to the community, where the community is needed to address and respond to the increasing burden of chronic diseases (35).

Effectiveness and Service delivery: Interview responses confirmed reduced waiting times and improved patient experience and satisfaction. Semi-automated dispensing improved operational efficiency, as the number of members and parcels increased over the years. Clinical governance strengthens the service through data-driven decision-making. Research in another province shows that patient waiting times and improving access improve the overall effectiveness of the CDU programme (36, 37). Sociodemographic factors, such as reduced waiting times and the number of clinic visits, are key indicators of patient satisfaction (38). Participants reported improvements in prescribing practices and more efficient dispensing workflows due to the reduction in facility congestion. There are several ongoing activities and improvements within the public health system to improve the overall service delivery, in preparation for NHI; however, the long-term impact is still unclear (39).

Adoption and Integration: Adoption of the CDU was initially slow, due to resistance and unfamiliarity with the CDU model. Rolled out across the Cape Town Metropole area; it eventually grew, supported by management, structured training and policy alignment. This mirrors findings from other health interventions, where strong leadership support, stakeholder engagement and capacity building were critical to a successful implementation (22, 24). This demonstrates that integrating an intervention requires organisational capacity, infrastructure, staffing, planning, and programme champions to facilitate the process.

Implementation challenges and innovations: Operational challenges were experienced, including insufficient infrastructure, space constraints, prescription errors and non-collection of medicine parcels. Fortunately, staff made innovative adaptations at the facility level, illustrating contextual tailoring. Hawe et al. (2009) described how successful adoption in resource-constrained settings depended on tailoring or customising current systems, like infrastructure, cultural norms, and workforce (40). This type of tailoring or innovation fosters ownership, ensures sustainability and subsequently increases the effectiveness of the intervention (23). The adaptations reflect the importance of local innovation and flexibility in sustaining health interventions, as highlighted in studies on integrated chronic care models. Shayo et al. (2023) demonstrated that adaptation, like reorganising facility spaces, combining services and using flexible models reflect the role of local innovation and responsiveness (41). Flexibility in design, delivery, and locally adapted strategies improved acceptability, continuity of medicine supply, and efficiency. A change in CDU service provider revealed weaknesses in data-handover and contract management, which were further clarified during subsequent contract negotiations. There is a proven need for stakeholder engagement and monitoring to identify outcomes and ensure accountability and responsiveness. The findings highlighted the need for continuous improvement mechanisms and robust governance structures, consistent with the implementation science literature (26).

Maintenance and Sustainability: The CDU is now embedded in routine operations of PHC facilities. The CDU's maturity of its system has strengthened maintenance and sustainability, though concerns remain about data integration, financial sustainability, and systemic challenges. The limited integration of CDU dispensing data with clinical information systems constrains the programme's success and its ability to inform population health outcomes. Similar data integration challenges are experienced in countries like Ghana (42) and Nigeria (43) as they strive toward integrated health information systems. Financial constraints, such as non-collection of medicine parcels, healthcare budgets and increased future service fees, threaten long-term sustainability. Participant feedback emphasised the need for improved patient selection to reduce the waste associated with the non-collection of parcels. Similar research documented barriers to medicine collection through the CCMDD programme in a different province (44). Whilst the CDU achieved high-volume dispensing of medicine parcels and improved access, non-collection of medicines or delayed returns of medicine parcels suggest variability in fidelity. These issues are more pronounced in the Cape Town Metropole area, where the scale of operations amplifies both the benefits and the risks.

### 4.2 Strengths and limitations

Strengths: The qualitative research used a robust method design grounded in both the RE-AIM and CFIR frameworks. This was done to explore the implementation and sustainability of the CDU programme in the WC. The research allowed for the examination of a real-world health intervention, ensuring that both contextual and process factors were addressed. The strength of the research lies in its purposive sampling strategy, whereby participants represented a broad spectrum of the health system, from primary care to operational and strategic management. This diversity enabled a comprehensive understanding of all aspects of service delivery, management processes and their system-level sustainability. Rigour was maintained through reflective practice, with an independent review of audio recordings and transcripts. This strengthened the credibility and dependability of the findings. Triangulation across participant perspectives enhanced its validity and provided a rich understanding of experiences and the determinants influencing the outcomes. Few studies explicitly used RE-AIM/CFIR frameworks to examine centralised dispensing at scale. The research provides an in-depth, contextually grounded exploration of the implementation process, rather than generalised performance metrics. Peer-reviewed works treated similar programmes, such as CCMDD, generically. Nonetheless, the consistency across participants and their alignment with the published literature support the trustworthiness and contribution of these findings to long-term health system interventions.

Limitations: The following limitation should be considered. The study has a small sample size, is purposively selected, and may not capture the full diversity of perspectives from other provinces. Interviews rely on participants’ willingness to disclose information, particularly when discussing sensitive or challenging topics. Thematic saturation was assessed iteratively during data collection and analysis. It was further refined as recurring themes emerged. Any additional data was unlikely to alter any of the core findings. However, the exploratory nature of the research and self-reported data limit the generalisability of its conclusions beyond this context. Since interviews and data were collected only once, it was influenced by recall or social bias. The identified facilitators and barriers may be unique to the current timeline. They may not reflect other points in time. Future evaluation should include fidelity measures such as the monitoring of parcel handling, patient tracing, and medication adherence to strengthen the impact and sustainability of the intervention (45). Finally, there is an absence of quantitative triangulation of patient-level outcome data due to the lack of linkage (data integration) between the dispensing and clinical records. This means the perceptions and experiences of the participants are stated, rather than empirical measures of the programme's effectiveness.

### 4.3 Implications and recommendations

#### 4.3.1 Policy and health system implications

The qualitative findings of the research highlight the important role of the CDU in the WC in providing equitable access to chronic medication and alleviating pressure at the hospitals and clinics. However, sustainability challenges highlight the need to strengthen health system integration. The focus should be on improving the interface between dispensing data, clinical data systems and the Provincial Health Data Centre (PHDC) to enable the monitoring of patient outcomes and enhance public health planning.

#### 4.3.2 Programme management and operational practice

At an operational level, stringer feedback loops should exist between the facilities, the CDU service provider and the WCGHW management to support continuous quality improvement. The introduction of digital patient tracking systems would facilitate proactive management of non-collection and adherence challenges. It is recommended that the WCGHW further improve the efficiency of the programme by improving patient selection into the CDU programme.

#### 4.3.3 Data and research implications

The CDU has had a profound impact on the WC's health system. Over the past two decades, it has evolved into a cornerstone of access to medicine in the region. However, the lack of integrated patient-level data remains a major limitation to assessing the programme’s impact on patient outcomes. Few peer-reviewed studies link centralised dispensing records to patient-level clinical outcomes in South Africa. Future research should incorporate mixed-method or longitudinal designs to quantify the effect of medicine collection and adherence to treatment, disease management and health outcomes.

#### 4.3.4 Broader implications for implementation science

The dual framework was valuable in identifying implementation success and challenges. Their use in the study supports the continued application in low- and middle-income settings. Future studies should include implementation fidelity specification, measurement and assessment of the interaction between context, process and outcomes to inform scalable and adaptive pharmaceutical and health interventions that will better inform funders, programme managers, clinicians, researchers and patients (45).

## 5 Conclusion and future research

Universal health care aims to improve access to PHC services, particularly for chronic conditions that require long-term management. As such, primary care plays a vital role in integrating chronic disease management into routine healthcare services, particularly within the framework of an integrated service model. This research demonstrates that the CDU has made a substantial contribution to improving equitable access to chronic medication and alleviating service pressures within the WC health system. However, challenges remain with the integration of data between the different systems, and the long-term sustainability continues to limit its impact on the health outcomes of patients. Future research should quantify the effect on clinical outcomes and identify factors that enhance scalability and sustainability. This will ensure that the CDU evolves into a data-driven and sustainable health system model for chronic disease management.

## Data Availability

The raw data supporting the conclusions of this article will be made available by the authors, without undue reservation.
